# An investigation into the relationship between plain water intake and glycated Hb (HbA1c): a sex-stratified, cross-sectional analysis of the UK National Diet and Nutrition Survey (2008–2012)

**DOI:** 10.1017/S0007114516003688

**Published:** 2016-11-10

**Authors:** Harriet A. Carroll, James A. Betts, Laura Johnson

**Affiliations:** 1 Department for Health, University of Bath, Claverton Down, BathBA2 7AY, UK; 2 School for Policy Studies, University of Bristol, 8 Priory Road, BristolBS8 1TZ, UK

**Keywords:** Fluid balance, Metabolism, Hydration, Glycated Hb, Type 2 diabetes

## Abstract

The aim of this study was to analyse the association between plain water intake and glycated Hb (HbA1c) in the National Diet and Nutrition Survey (2008–2012) rolling survey. These data included diet (4-d diaries) and HbA1c (fasted blood sample) measures of 456 men and 579 women aged 44 (sd 18) years with full information on covariates of interest (age, ethnicity, BMI, smoking status, education, other beverage intake, energy intake and fibre). Data were analysed using sex-stratified linear and logistic regressions modelling the associations of cups per d (240 ml) of plain water with HbA1c, and odds of HbA1c≥5·5 %, respectively. Substitution analyses modelled the replacement of sugar-sweetened beverages, fruit juice and artificially sweetened beverages with plain water. After adjustment, 1 cup/d of plain water was associated with a −0·04 % lower HbA1c (95 % CI −0·07, −0·02) in men. In logistic regression, men had a 22 % (95 % CI 10, 32 %) reduced odds of HbA1c≥5·5 %/cup per d of plain water. There was no evidence of an association with either HbA1c or odds of HbA1c≥5·5 % in women. None of the substitution models was associated with a change in odds of HbA1c≥5·5 %. Plain water intake was associated with lower HbA1c in men but not in women. Substituting water for specific beverages was not associated with a reduced odds of HbA1c≥5·5 %, suggesting that the addition of water is the more pertinent factor. Future trials should test whether the relationships between water intake and HbA1c is causal as this could be a cost-effective and simple health intervention.

It is commonly recommended to drink water as part of a healthy diet; yet, optimal intakes are unknown, meaning guidelines often refer to adequate intakes^(^ ^1^ ^)^. Water is essential for normal metabolism and may also specifically be associated with reduced type 2 diabetes (T2D) risk; in brief, in addition to plain water intake being a marker of a generally healthier lifestyle (associated with lower sugar intake and more physical activity (PA)^(^ ^2^ ^)^), and potentially increasing satiation^(^ ^3^ ^)^, it also contributes to hydration. This reduces arginine vasopressin secretion^(^ ^4^ ^,^ ^5^ ^)^ (AVP, a blood pressure regulating hormone that impacts glycaemia) and increases plasma volume (subsequently decreasing the plasma concentration of glucose^(^ ^6^ ^)^), both of which influence glucose homoeostasis^(^ ^5^ ^,^ ^6^ ^)^.

There is also evidence that osmolality (which can be influenced by hydration status and is a key influencer of AVP secretion) impacts glucose metabolism; higher plasma glucose concentrations have been found during hyperosmolality compared with iso-osmolality or hypo-osmolality^(^ ^7^ ^)^, supported by an increase in hepatic gluconeogenesis during hyperosmolality^(^ ^7^ ^)^ and dehydration^(^ ^6^ ^)^. Extracellular osmolality also impacts cell volume and intracellular metabolism, which may explain these findings possibly due to the role of insulin on cell swelling and glucagon on cell shrinkage^(^ ^7^ ^,^ ^8^ ^)^. These potential mechanisms linking water intake to glycaemic control are outlined in Fig. 1.

**Fig. 1:**
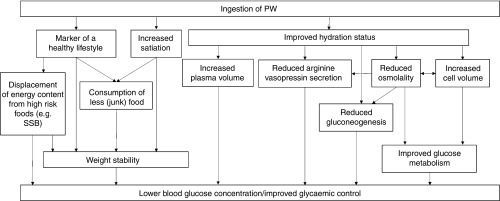
Mechanisms potentially associating increased water intake with improved glycaemic control. Ingestion of plain water (PW) is a marker of a healthy lifestyle^(^ ^2^ ^)^; part of this can manifest itself in consuming PW in place of unhealthy foods/beverages or by coincidental consumption of less unhealthy food. Further, PW intake is debatably linked to increased satiation^(^ ^3^ ^)^, thus potentially reducing energy consumption. These factors contribute to improved glycaemia both indirectly (via weight stability) and directly (via fewer and/or lower blood glucose concentration spikes). Ingestion of PW also contributes to hydration. Improved hydration status reduces the secretion of arginine vasopressin (a blood pressure regulating hormone that plays an important role in glycaemic control)^(^ ^4^ ^,^ ^5^ ^)^. Having an improved hydration status increases plasma volume, which could reduce the concentration of blood glucose^(^ ^6^ ^)^. Finally, hydration status directly impacts osmolality, which in turn impacts arginine vasopressin secretion. Hydration also increases cell volume^(^ ^8^ ^)^, which both impacts and is impacted by osmolality. Both these factors effect cellular glucose metabolism^(^ ^5^ ^,^ ^6^ ^)^, resulting improved glycaemic control when euhydrated. Further, both dehydration^(^ ^6^ ^)^ and higher osmolality increase hepatic gluconeogenesis^(^ ^7^ ^)^, which may negatively affect glycaemia. SSB, sugar-sweetened beverages.

Despite plausible mechanisms, there is a paucity of research directly investigating the relationship between plain water intake and glycaemia. Although some studies have included water intake as a secondary finding or substitution^(^ ^9^ ^,^ ^10^ ^)^, only three have directly investigated the relationship between water intake and T2D risk or hyperglycaemia. In the US Nurses’ Health Study II (NHS-II), plain water intake was not associated with a change in T2D risk, across five intake categories ranging from <1 cup/d (reference category) to ≥6 cups/d^(^ ^11^ ^)^. In the multivariable model, consuming 1 or 2–3 cups/d was non-significantly associated with a 7 % lower T2D risk, whereas consuming 4–5 or ≥6 cups/d was associated with a non-significant 6–9 % increased risk of T2D compared with the reference category^(^ ^11^ ^)^.

Conversely, in the Data from Epidemiological Study on Insulin Resistance Syndrome cohort in France, after adjustment for potential confounders, 0·5–1 litres/d of plain water was associated with a significantly lower odds of hyperglycaemia compared with consuming <0·5 litres/d (OR 0·68; 95 % CI 0·52, 0·89)^(^ ^4^ ^)^. Consuming >1 litres/d was also associated with lower odds compared with consuming <0·5 litres/d, although this was not significant (OR 0·79; 95 % CI 0·59, 1·05)^(^ ^4^ ^)^. A small cross-sectional study in the UK found that 1 cup (240 ml)/d of plain water was linearly associated with a 0·72-point reduced T2D risk (based on a 0–47-point scale of T2D risk characteristics)^(^ ^12^ ^)^.

The results of existing studies are somewhat conflicting, potentially explained by the validity of the dietary data used (FFQ including plain water intake, with two studies reporting no validation^(^ ^4^ ^,^ ^12^ ^)^), the characteristics of the cohorts used (nurses only^(^ ^11^ ^)^, convenience sampling^(^ ^12^ ^)^ or volunteers offered free health checks^(^ ^4^ ^)^) or by sex differences – Pan *et al*.^(^ ^11^ ^)^ included only females (finding no significant association between plain water intake and T2D risk), whereas the other studies^(^ ^4^ ^,^ ^12^ ^)^ included both sexes (finding an inverse association).

Plain water intake represents a potentially efficacious target for health promotion, which is simultaneously simple to understand and inexpensive. Cost-effective interventions are of particular importance as those from lower socio-economic statuses are at higher risk of metabolic diseases^(^ ^13^ ^)^. The present study therefore investigated the role of plain water intake on glycated Hb (HbA1c) in a large UK sample, hypothesising that plain water intake is associated with lower HbA1c. As a previous study was conducted only in women^(^ ^11^ ^)^ with conflicting results compared with other mixed-sex studies, we aimed to investigate whether there were differences between sexes by conducting sex-stratified analyses. Our final objective was to explore the influence of substituting certain beverages for plain water on HbA1c.

## Methods

### Study design and population

Full details of data collection are available online^(^ ^14^ ^)^. In summary, the National Diet and Nutrition Survey (NDNS) is a cross-sectional rolling survey that collects lifestyle and 4-d dietary records from approximately 1000 nationally representative respondents from the UK (aged 1·5–64 years) per year (2008–2012, *n* 4165). All respondents provided informed consent, and the study gained ethical approval from appropriate Local Research Ethics Committees^(^ ^14^ ^)^.

**Fig. 2:**
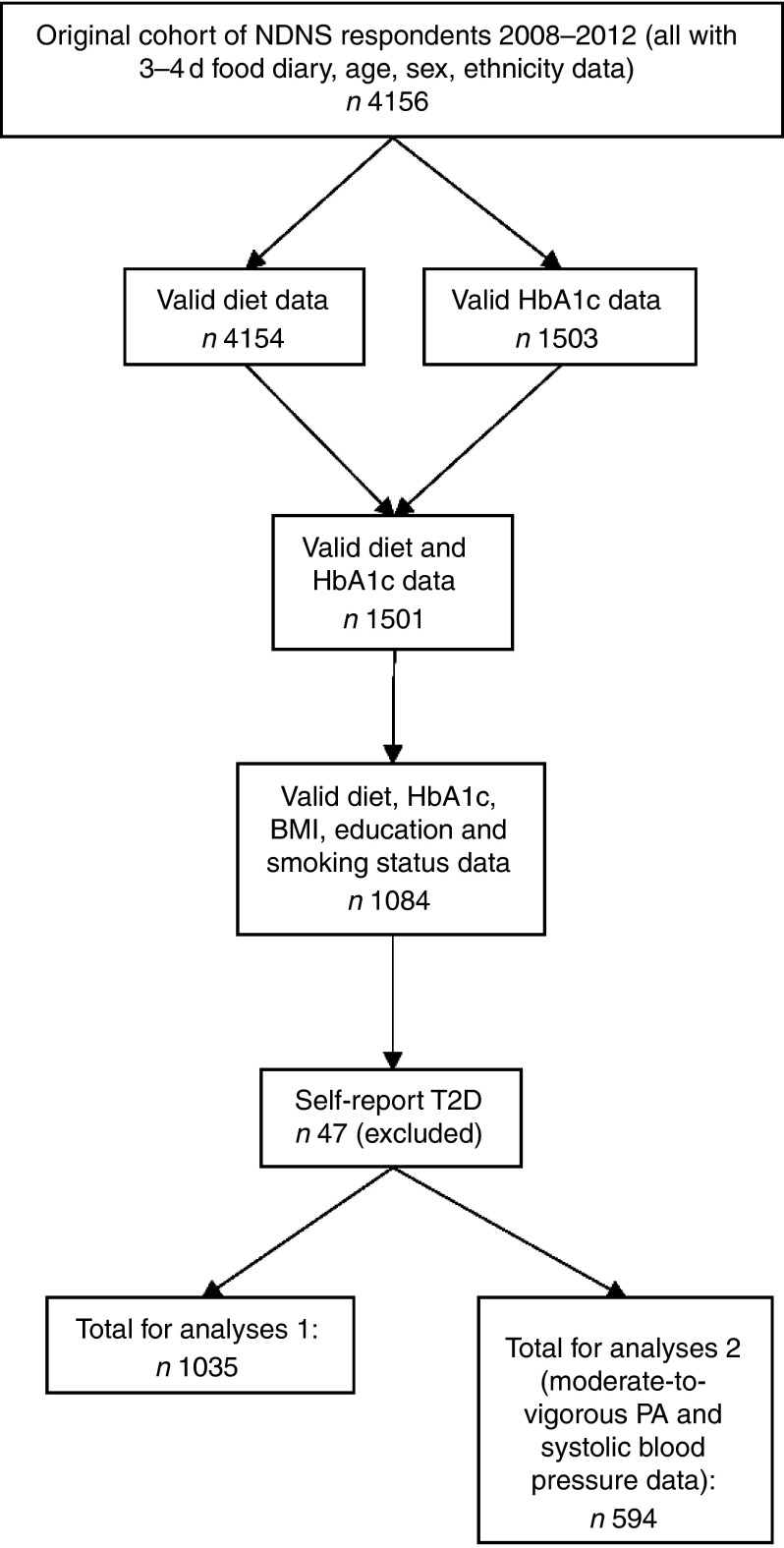
Flow chart of participant inclusion. NDNS, National Diet and Nutrition Survey; HbA1c, glycated Hb; PA, physical activity; T2D, type 2 diabetes.

In the present analyses, participants were included if they were aged ≥16 years (as they could opt-in for the blood test at this age), with diet/beverage and blood measures, full information on covariates of interest and without self-reported T2D diagnosis (Fig. 2). Those with self-reported T2D diagnosis were excluded in order to reduce the likelihood of reverse causality as diagnosis may lead to lifestyle change or a reduction in HbA1c in response to medication. The current analyses therefore used 1035 respondents’ data from the NDNS 2008–2012 data set and was approved by the Research Ethics Approval Committee for Health at the University of Bath (ref. no. EP 14/15 215). Data were downloaded from the UK Data Service^(^ ^15^ ^)^.

### Measure of diet

The NDNS protocol consists of 4-d unweighted food diaries (although some participants only provided 3-d data), with instructions on how to estimate portion size^(^ ^16^ ^)^. The NDNS data contain a breakdown of nutrients in each food consumed, including the water content of foods and beverages, based on the Department for Health NDNS Nutrient Databank^(^ ^16^ ^)^.

Plain water was the main exposure of interest. This was defined as water with no added flavours, non-nutritive sweeteners, nutrients (such as vitamins), stimulants or energy (Table 1). Thus, carbonated water was included as it fitted the above criteria with the only key difference being a slight increase in acidity compared with most bottled or tap waters.

**Table 1:** Description of beverage categories

Beverage groups	Description
Alcoholic beverages	Beer, wine, spirits, other drinks containing alcohol
Artificially sweetened beverages/low-energy beverages	Any beverage containing artificial sweeteners, including milk-based drinks, drinks with and without caffeine or any diet/reduced-energy soft drink
Fruit juice	100 % fruit or vegetable juice including from concentrate, but not with added sugar
Milk	Fully reduced and low-fat milks, including powdered varieties, but not hot chocolate, non-cow animal milks or plant-based milks
Plain water	Plain water with no added flavours (including non-nutritive sweeteners), nutrients (such as vitamins), stimulants or energy
Sugar-sweetened beverages	Carbonated and non-carbonated soft drinks with added sugar as sweetener, including fruit juices with added sugar, drinks with and without caffeine, and sugar-sweetened water beverages
Tea and coffee	Caffeinated and non-caffeinated, including green/other teas
Miscellaneous beverages	Hot chocolate, smoothies, water with additives such as Ca or vitamins, non-cow milks, plant-based milks, protein drinks

Other beverages were divided into beverage categories (Table 1), and water intake from each category was calculated. This was carried out by aggregating the appropriate food numbers and/or food groups from the ‘food level dietary data’ NDNS file, followed by adding up the water content of each category for each person accordingly. This figure was divided by the number of days of diet reported by each participant to provide average intake per day (g). Average cups (240 ml) per day of water from each beverage source were then calculated, as well as total water from food, and total water (water from plain water, plus beverages, plus food). Although the data are given in grams, the conversion from grams to millilitres for water is 1:1.

In order to account for misreporting of energy intake (EI) and indicate the completeness of the diet record, estimated energy requirements (EER) were calculated as per the Institute of Medicine^(^ ^17^ ^)^ equations (page 182 for males and females aged ≤18 years and page 185 for males and females aged ≥19 years^(^ ^17^ ^)^). For these purposes, those with no PA data were categorised as ‘sedentary’ as this was the modal average of the sample (based on self-report data).

Reported EI should equal EER under the assumption of energy balance; therefore, EI:EER=1. Because of the error in EI and EER estimations, we calculated CI for the EI:EER ratio, within which respondents were considered as ‘plausible’ reporters. To calculate the total CV, the equations and data from Black & Cole^(^ ^18^ ^)^, Institute of Medicine^(^ ^17^ ^)^ and Mendez *et al*.^(^ ^19^ ^)^ were used, with the CV of EI calculated using the individualised method^(^ ^20^ ^)^. In this sample, 73·2 % were deemed as under-reporters (mean EI:EER 0·63 (sd 0·14)), 21·4 % were plausible reporters (mean EI:EER 0·98 (sd 0·8)) and 5·4 % were over-reporters (mean EI:EER 1·28 (sd 0·16)), similar to a previous study using NDNS data^(^ ^20^ ^)^. Participants were then categorised according to their reporter category, which was included in the model as a confounder^(^ ^21^ ^)^.

### Measure of glycated Hb

Participants could opt-in for a blood test, which typically took place ≥8 weeks after diet recording. HbA1c was the outcome of interest. In order to obtain the sample, overnight fasted blood samples (33 ml) were drawn into EDTA tubes, and posted by the nurses to the UK National Health Service Laboratory at Addenbrooke’s Hospital, Cambridge, UK. Duplicates were run only if the results were outside the acceptable range^(^ ^22^ ^)^.

### Covariates

Lifestyle information was gained via interviews. This included PA (using the Recent Physical Activity Questionnaire, which was validated to estimate energy expenditure in the NDNS^(^ ^23^ ^)^), general dietary habits and other lifestyle factors (e.g. smoking status and highest level of education achieved)^(^ ^14^ ^)^ Objectively measured anthropometrics were also taken^(^ ^24^ ^)^.

### Statistical analysis

Descriptive statistics summarise the data according to tertiles of plain water intake; summaries according to HbA1c categories can be found in the online Supplementary Material. For the purposes of these summary statistics, three HbA1c categories were created, on the basis of a criterion for diagnosing T2D (≥6·5 %)^(^ ^25^ ^)^, increased cardiometabolic risk (5·5–6·49 %)^(^ ^26^ ^,^ ^27^ ^)^ and low cardiometabolic risk (<5·5 %)^(^ ^26^ ^–^ ^28^ ^)^. Data are presented as percentage of total, means and standard deviations or median and interquartile range (IQR) for each covariate, as appropriate. Differences between groups were tested using the *χ* ^2^ test, ANOVA with *post hoc* Bonferroni correction or the Kruskal–Wallis test with *post hoc* Dunn–Bonferroni correction as appropriate.

For beverage categories, the median (IQR) intake of consumers was calculated. To establish the relationship with plain water and HbA1c, correlations were run using Spearman’s *ρ*. The percentage contribution to total average daily water intake from different sources of water was calculated for binary HbA1c categories with <5·5 % (minimal risk^(^ ^26^ ^–^ ^28^ ^)^) and ≥5·5 % (increased risk^(^ ^26^ ^,^ ^27^ ^)^). Missing data analyses were conducted as above on respondents aged ≥16 years and are presented in the online Supplementary Material. Comparisons between included and excluded respondents were tested statistically using *t* test, Mann–Whitney *U* test or *χ* ^2^ test.

Potential confounding and mediating variables were identified from a literature search of studies on T2D and beverages as well as theory. The following confounders were identified for inclusion in the model and were available in the NDNS data set: age, ethnic group (white or non-white), BMI ( kg/m^2^), smoking status (current smoker or not), education level (holds a degree or not), total average daily water from beverages minus average daily plain water intake, systolic blood pressure (mmHg) and PA (h/d moderate-to-vigorous PA).

In addition, two mediator models were included. EI (kJ (kcal)) and reporter category (under-, plausible- or over-reporter) were included in one model, and fibre (g/4184 kJ (g/1000 kcal)) was included in another model. Although reporter category is technically a confounder, it was included in the mediator model of EI in order to account for misreporting in the same model. Missing data were not imputed (this was only conducted to calculate the EI:EER). Thus, if adding a covariate substantially reduced the sample size, as in the case of PA and systolic blood pressure, a new analysis was run in the smaller sample.

Multiple linear regression using heteroscedasticity-consistent standard error estimators^(^ ^29^ ^)^ was conducted with continuous HbA1c as the outcome variable. Model assumptions were checked and met. Linearity was formally tested using nested regression models and compared using a likelihood ratio test.

Logistic regression was conducted to test differences between those at low (HbA1c<5·5 %^(^ ^26^ ^–^ ^28^ ^)^) and increased (HbA1c≥5·5 %^(^ ^26^ ^,^ ^27^ ^)^) cardiometabolic risk in order to provide clinically meaningful results. Model assumptions were checked and met. For both regressions, systolic blood pressure (mmHg) and moderate-to-vigorous PA (h/d) were added in a separate model, because of the loss in sample size (outlined in Fig. 2). All analyses were split by sex based on sex differences noted when comparing previous studies^(^ ^4^ ^,^ ^11^ ^,^ ^12^ ^)^. Sex differences in the association between plain water intake and HbA1c were confirmed statistically by adding an interaction term into the models (linear regression sex×plain water intake interaction *P*=0·010; logistic regression sex×plain water intake interaction *P*=0·008).

Finally, four substitution models were run in order to estimate the association with HbA1c of substituting one beverage for another. The effect estimates were generated using logistic regression with the same covariates as above but with all drink categories included in the model individually (rather than as total beverages). The following substitutions were conducted, using the post-estimation command *lincom* in Stata, which linearly combines the exp(b) values for each drink and estimates the 95 % CI based on the variance and covariance of both drinks: substituting sugar-sweetened beverages (SSB) for plain water, substituting fruit juice for plain water, substituting artificially sweetened beverages (ASB) for plain water and substituting SSB for ASB. The final substitution was run in order to find whether an alternative no/low-energy beverage would be associated with a better HbA1c profile, as adherence to this substitution may be easier compared with plain water^(^ ^30^ ^)^.

Data were not weighted to take into account known socio-demographic differences between responders and non-responders, as previous studies have shown the impact of this adjustment to be extremely small and not significant^(^ ^31^ ^–^ ^33^ ^)^. In addition, we were only interested in the relationship between variables rather than estimates of disease prevalence^(^ ^32^ ^)^. For all analyses, the *α* level was set at ≤0·05 (two-tailed) and were run using SPSS (version 22; IBM), except for the substitution models, which were run in Stata (version 13; StataCorp LP), as necessary for completing the respective analyses.

## Results

A total of 1035 respondents were included in these analyses (Fig. 2). Of the sample, 44 % were men. The mean age and BMI were 44 (sd 19) years and 26·7 (sd 4·6) kg/m^2^ among men (Table 2) and 46 (sd 18) years and 27·0 (sd 5·9) kg/m^2^ among women (Table 3), respectively. In this study, 76 % of men and 88 % of women reported consuming plain water. The median intake was 1·4 (IQR 0·5, 2·6) cups/d in men and 1·5 (IQR 0·7, 2·8) cups/d in women (Table 4). Mean HbA1c was 5·5 (SD 0·5) % for men and 5·5 (SD 0·4) % for women (Tables 2 and 3, respectively).

**Table 2:** Characteristics of male National Diet and Nutrition Survey respondents according to plain water intake (*n* 456) (Mean values and standard deviations/interquartile ranges (IQR))

	Plain water intake								
Characteristics	Total	SD/IQR	≤0·21 cups/d	SD/IQR	0·22–1·59 cups/d	SD/IQR	>1·59 cups/d	SD/IQR	*P*_for difference_
Participants (*n*)	456	–	148	–	155	–	153	–	–
Ethnicity (% white)	93	–	98	–	94	–	88	–	0·003
Age (years) (mean)	44	19	46	19	47	19	39	17	<0·001*†
HbA1c (% mean)	5·5	0·5	5·6	0·6	5·5	0·4	5·3	0·4	<0·001*†
Current smoker (%)	22	–	25	–	21	–	20	–	0·545
BMI (kg/m^2^ mean)	26·7	4·6	26·4	4·7	27·0	4·2	26·6	4·8	0·463
Education (% degree or more)	23	–	18	–	21	–	29	–	0·043
EI (kJ/d mean)	8987	2481	8606	2460	9017	2259	9322	2674	
EI (kcal/d mean)	2148	593	2057	588	2155	540	2228	639	0·043*
EI:EER (mean)	0·73	0·23	0·71	0·23	0·75	0·23	0·74	0·23	0·425
Fibre (g/4184 kJ (g/1000 kcal) mean)	7	2	7	2	7	2	8	2	0·019*
Participants (*n*)	258	–	92	–	88	–	78	–	–
Systolic blood pressure (mmHg mean)	130	14	128	14	132	14	130	14	0·257
MVPA (h/d median)‡	1·2	0·5, 3·1	0·9	0·3, 3·0	1·1	0·5, 3·4	1·5	0·8, 3·2	0·073

HbA1c, glycated Hb; EI, energy intake; EER, estimated energy requirement; MVPA, moderate-to-vigorous physical activity. 1 cup=240 ml. * Significantly different between ≤0·21 and >1·59 cups/d (*P*≤0·037). † Significantly different between 0·22–1·59 and >1·59 cups/d (*P*≤0·001).

‡ Differences calculated using Kruskal–Wallis with *post hoc* Dunn–Bonferroni correction; all other differences calculated using ANOVA with *post hoc* Bonferroni correction.

**Table 3:** Characteristics of female National Diet and Nutrition Survey respondents according to plain water intake (*n* 579) (Mean values and standard deviations/interquartile ranges (IQR))

	Plain water intake								
Characteristics	Total	SD/IQR	≤0·66 cups/d	SD/IQR	0·67–2·05 cups/d	SD/IQR	>2·05 cups/d	SD/IQR	*P*_for difference_
Participants (*n*)	579	–	194	–	192	–	193	–	–
Ethnicity (% white)	93	–	95	–	89	–	94	–	0·044
Age (years) (mean)	46	18	46	17	48	19	45	18	0·200
HbA1c (% mean)	5·5	0·4	5·5	0·4	5·5	0·4	5·4	0·4	0·050*
Current smoker (%)	18	–	22	–	17	–	14	–	0·079
BMI (kg/m^2^ mean)	27·0	5·9	26·7	5·8	26·7	5·8	27·6	6·1	0·233
Education (% degree or more)	23	–	14	–	24	–	30	–	0·001
EI (kJ/d mean)	6745	1774	6540	1753	6837	1787	6862	1770	
EI (kcal/d mean)	1612	424	1563	419	1634	427	1640	423	0·135
EI:EER (mean)	0·75	0·23	0·74	0·23	0·77	0·24	0·75	0·22	0·214
Fibre (g/4184 kJ (g/1000 kcal) mean )	8	3	8	3	8	3	9	3	0·050**
Participants (*n*)	336	–	113	–	109	–	114	–	–
Systolic blood pressure (mmHg mean)	122	17	122	16	122	18	122	16	0·999
MVPA (h/d median)†	0·6	0·3, 1·3	0·4	0·1, 1·0	0·7	0·3, 1·5	0·8	0·3, 1·5	0·002***

HbA1c, glycated Hb; EI, energy intake; EER, estimated energy requirement; MVPA, moderate-to-vigorous physical activity. 1 cup=240 ml.

* Significantly different between 0·67–2·05 and >2·05 cups/d (*P*≤0·048).

** No significant difference between any group after correction.

*** Significantly different between ≤0·66 and 0·67–2·05 cups/d (*P*=0·019) and significantly different between ≤0·66 and ≥2·05 cups/d (*P*=0·003).

† Differences calculated using Kruskal–Wallis with *post hoc* Dunn-Bonferroni correction; all other differences calculated using ANOVA with *post hoc* Bonferroni correction.

**Table 4:** Water from difference sources and association with plain water (PW) intake and glycated Hb (HbA1c) (Medians and interquartile ranges (IQR))

	Men (*n* 456)					Women (*n* 579)				
Beverage types	Consumers (%)†	Median intake†	IQR	Correlation with PW	Correlation with HbA1c	Consumers (%)†	Median intake†	IQR	Correlation with PW	Correlation with HbA1c
PW (cups/d)	76	1·4	0·5, 2·6	–	−0·25**	88	1·5	0·7, 2·8	–	−0·05
Alcoholic drinks (cups/d)	60	1·7	0·5, 3·1	−0·02	−0·08	55	0·5	0·2, 1·0	0·04	−0·14*
ASB (cups/d)	32	0·7	0·3, 1·9	−0·09	−0·05	38	0·8	0·3, 1·7	−0·04	−0·06
Fruit juice (cups/d)	41	0·4	0·2, 0·7	−0·06	0·06	41	0·3	0·1, 0·6	−0·04	0·04
Milk (cups/d)	93	0·6	0·3, 1·0	−0·06	0·14**	95	0·5	0·3, 0·8	−0·09*	0·11*
SSB (cups/d)	52	0·9	0·4, 1·6	0·03	0·01	50	0·5	0·3, 1·0	0·00	0·21**
Tea/coffee (cups/d)	88	3·0	1·7, 4·1	−0·15**	0·21**	93	3·0	1·9, 4·2	−0·12**	0·26**
Miscellaneous beverages (cups/d)	16	0·3	0·2, 0·8	−0·12	−0·07	21	0·5	0·2, 0·8	0·02	0·13
Total beverages (cups/d)‡	100	5·8	4·1, 7·7	−0·17**	0·06	100	4·9	3·5, 6·4	−0·12**	0·17**
Water from food (g/d)	100	579	458, 739	0·20***	0·05§	100	528	410, 654	0·22***	0·12**§
Total water (g/d)‡	100	2015	1571, 2546	−0·09	0·02§	100	1729	1329, 2147	−0·04	0·18**§

ASB, artificially sweetened beverages; SSB, sugar-sweetened beverages. 1 cup=240 ml. * *P*<0·05, ** *P*<0·01, *** *P*<0·001.

† Percentage of the sample who reported consuming each beverage type; median intakes are of consumers only.

‡ Total minus PW.

§ Pearson’s correlation used; all other correlations used Spearman’s *ρ*.

In unadjusted analyses, HbA1c was lower in men consuming ≤0·21 and 0·22–1·59 cups/d of plain water compared with >1·59 cups/d (*P*≤0·037 and *P*≤0·001, respectively; Table 2). Similarly, women in the middle tertile (consuming 0·67–2·05 cups/d) of plain water consumption had higher HbA1c compared with those in the upper tertile (consuming >2·05 cups/d; *P*=0·048; Table 3). In both men (*P*=0·462) and women (*P*=0·233), BMI did not vary according to plain water intake. Participants with at least a degree were more likely to consume higher amounts of plain water among men (*P*=0·043; Table 2) and women (*P*=0·001; Table 3). Systolic blood pressure was higher in those with HbA1c≥6·5 % in men (*P*≤0·016; online Supplementary Table S1) and HbA1c≥5·5 % in women (*P*≤0·033; online Supplementary Table S2), compared with those with HbA1c<5·5 %.

Water intake from different beverage sources and the relation with plain water intake and HbA1c are shown in Table 4. SSB were consumed in larger quantities among male consumers, but were positively correlated with HbA1c in females only (*r*_s_ 0·21). Milk intake was positively correlated with HbA1c in men (*r*_s_ 0·15) and women (*r*_s_ 0·11), as were tea and coffee intakes (*r*_*s*_ 0·21, *r*_s_ 0·26 for men and women, respectively). Tea and coffee were also negatively correlated with plain water intake (*r*_s_ −0·15 for men and *r*_s_ −0·12 for women; Table 4).

**Fig. 3:**
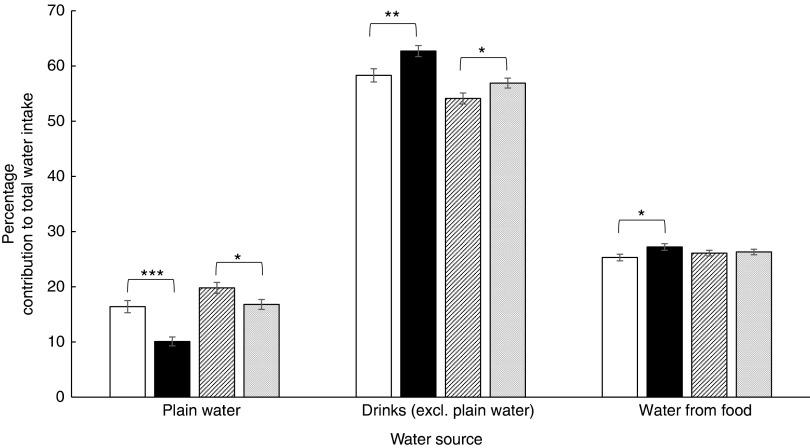
Percentage contribution of different sources of water by glycated Hb (HbA1c) categories (low cardiometabolic risk <5·5 %^(^ ^26^ ^–^ ^28^ ^)^; increased cardiometabolic risk 5·5–6·49 %^(^ ^26^ ^–^ ^27^ ^)^). Values are means, with their standard errors represented by vertical bars. Differences tested using Kruskal–Wallis test. * The difference in intakes between HbA1c categories is significant (*P*<0·05). ** The difference in intakes between HbA1c categories is significant (*P*=0·007). *** The difference in intakes between HbA1c categories is significant (*P*<0·001). 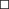, Men <5·5 % (*n* 221); 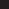, men ≥5·5 % (*n* 235); 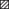, women <5·5 % (*n* 295); 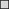, women ≥5·5 % (*n* 284). Excl., excluding.

Those with HbA1c<5·5 % had a greater contribution to total water from plain water in both men (*P*<0·001) and women (*P*<0·05; Fig. 3). In addition, this group (HbA1c<5·5 %) had a lower contribution from other drinks in both men (*P*=0·007) and women (*P*<0·05; Fig. 3). Although there was no significant difference in the contribution from water in food in women, men with HbA1c<5·5 % consumed significantly less water from food than men with HbA1c≥5·5 % (*P*<0·05; Fig. 3).

**Fig. 4:**
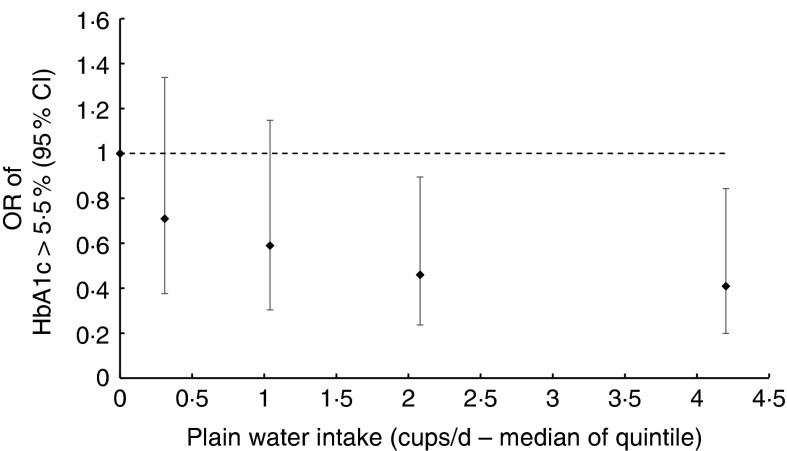
OR of glycated Hb (HbA1c) ≥5·5 % according to median plain water intake of each quintile of consumption in men. The likelihood ratio test suggested no evidence of deviation from linearity (*P*=0·600), calculated by comparing nested regression models.

**Fig. 5:**
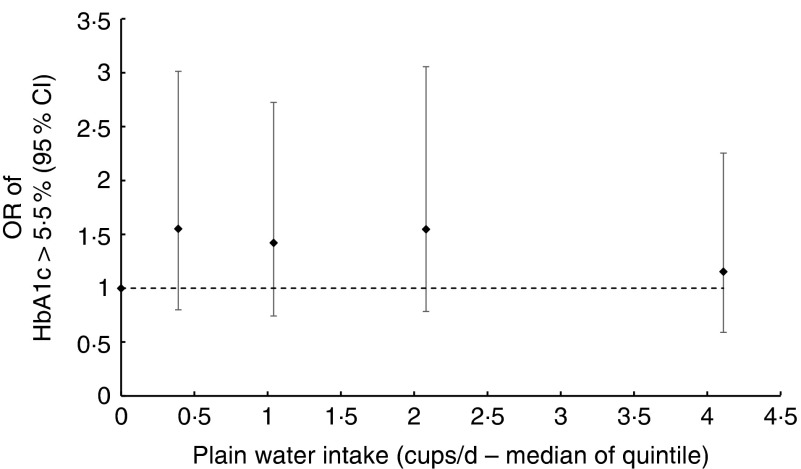
OR of glycated Hb (HbA1c) ≥5·5 % according to median plain water intake of each quintile in women. The likelihood ratio test suggested no evidence of deviation from linearity (*P*=0·451), calculated by comparing nested regression models.

Likelihood ratio tests of nested regression models provided no evidence of deviation from a linear trend (*P*=0·451 men, *P*=0·600 women; Fig. 4 and 5, respectively). In the unadjusted linear regression analysis, 1 cup/d of plain water was associated with lower HbA1c (*B* −0·05 %; 95 % CI −0·08, −0·02; Table 5) in men. After adjusting for age, ethnic group, BMI, smoking status and education level, this was attenuated slightly to −0·03 % (95 % CI −0·06, −0·01). After further adjustment for total drinks, EI, reporter category and fibre intake, these coefficients altered slightly (*B* −0·04; 95 % CI −0·07, −0·02). In the unadjusted model for women, 1 cup/d plain water was associated with −0·15 % (95 % CI −0·17, −0·13) lower HbA1c. However, after further adjustment (model 2), there was no evidence of lower HbA1c per cup/d plain water, which remained unchanged in the most adjusted model (*B* −0·01; 95 % CI −0·02, 0·01; Table 5). Including PA and systolic blood pressure reduced the sample size (men *n* 258; women *n* 336), but did not meaningfully alter the coefficients in the linear regression models (online Supplementary Table S3).

**Table 5:** Linear regression analysis of cups per day of plain water on glycated Hb (HbA1c) in men (*n* 456) and women (*n* 579)* (*B* values and 95 % confidence intervals)

	Men			Women		
Models†	*B* ‡	95 % CI	*P*_for trend_	*B* ‡	95 % CI	*P*_for trend_
1	−0·05	−0·08, −0·02	<0·001	−0·15	−0·17, −0·13	0·111
2	−0·03	−0·06, −0·01	<0·001	−0·01	−0·02, 0·01	0·293
3	−0·04	−0·06, −0·01	<0·001	−0·01	−0·02, 0·01	0·346
4	−0·04	−0·07, −0·02	<0·001	−0·01	−0·02, 0·01	0·326
5	−0·04	−0·07, −0·02	<0·001	−0·01	−0·02, 0·01	0·313

* *P*_for interaction_=0·010 for HbA1c and water intake between sexes.

† Model 1=plain water; model 2=model 1+age, ethnic group, BMI, smoking status, qualifications (degree or not); model 3=model 2+total drinks minus plain water (g); model 4=model 3+energy intake, reporter category; model 5=model 4+fibre (g/4184 kJ (g/1000 kcal)).

‡ *B* value represents the change in HbA1c per increase of 1 cup/d of water.

The unadjusted logistic regression model showed that 1 cup/d plain water significantly reduced the odds of HbA1c≥5·5 % by 22 % (OR 0·78; 95 % CI 0·69, 0·87) in men, which did not meaningfully change after subsequent adjustment for age, ethnicity, BMI, smoking status, qualification status, total drinks, EI, reporter category and fibre intake (Table 6). No association was found for women, including after adjusting for covariates (in the most adjusted model OR 0·98; 95 % CI 0·88, 1·08). After including PA and systolic blood pressure into the model (thus reducing the sample size), the OR altered, with the most notable difference resulting in no significant change in odds of HbA1c≥5·5 %/cup per d plain water in men (online Supplementary Table S4). Finally, substituting SSB, fruit juice or ASB for plain water did not significantly reduce the odds of having HbA1c≥5·5 % in men or women, nor did substituting SSB for ASB (online Supplementary Table S5).

**Table 6:** Logistic regression of cups per day of water on glycated Hb (HbA1c) ≥5·5 % compared with <5·5 % in men (*n* 456) and women (*n* 579)* (Odds ratios and 95 % confidence intervals)

	Men		Women	
Models†	OR	95 % CI	OR	95 % CI
1	0·78	0·69, 0·87	0·94	0·86, 1·03
2	0·79	0·70, 0·90	0·97	0·88, 1·08
3	0·78	0·68, 0·88	0·98	0·89, 1·09
4	0·76	0·67, 0·87	0·98	0·89, 1·09
5	0·78	0·68, 0·90	0·98	0·88, 1·08

* *P*_for interaction_=0·008 for HbA1c and water intake between sexes.

† Model 1=plain water; model 2=model 1+age, ethnic group, BMI, smoking status, qualifications (degree or not); model 3=model 2+total drinks minus plain water (g); model 4=model 3+energy intake, reporter category; model 5=model 4+fibre (g/4184 kJ (g/1000 kcal)).

Missing data analysis (online Supplementary Table S6 and S7) showed that the sample analysed in this study was more physically active and had a higher EI compared with excluded respondents, but with no significant difference in BMI compared with those who were excluded. In addition, included women were more likely to have a degree and less likely to currently smoke than excluded women. Despite this, overall beverage consumption trends were fairly consistent between included and excluded respondents (online Supplementary Table S8). Differences were that more included than excluded women consumed alcohol, tea/coffee and milk, leading to higher overall beverage consumption and total water intake. Included men consumed more tea/coffee than excluded men, although there were no significant differences in total beverages or total water intake.

## Discussion

In this cross-sectional analysis of 1035 adults in the UK NDNS, we found that higher plain water intake was associated with lower HbA1c in men, but not in women, independent of a range of confounders. Of note is that the inclusion of PA and systolic blood pressure in the logistic regression model resulted in no significant change in odds of HbA1c≥5·5 %/cup per d plain water in men. The lack of statistical significance compared with the models excluding PA and systolic blood pressure is likely a result of the reduction in sample size compromising the power to detect the small effect size found.

A 0·04 % lower in HbA1c was found per cup per day of plain water consumed in men. The Food and Drug Administration^(^ ^34^ ^)^ and the European Medicines Agency^(^ ^35^ ^)^ class a reduction of 0·3 % as clinically meaningful. These analyses therefore show that 1 cup/d of water did not contribute to clinically meaningfully lower HbA1c, despite reaching statistical significance. In saying this, on a population level, small changes can notably improve public health. Thus, if these results are confirmed in causal research, increasing water intake may be a viable intervention to improve population-level cardiometabolic health.

Due to the imperfect measure of dietary data, the potential for reverse causation in the cross-sectional study design and the inability to fully control for residual confounding factors, these results should be interpreted cautiously. However, our findings are in accordance with previous epidemiological studies. Roussel *et al*.^(^ ^4^ ^)^ found a 32 % lower risk of hyperglycaemia in those consuming 0·5–1 litres/d plain water compared with <0·5 litres/d. Risk was non-significantly lower at higher intake (>1 litres/d). The non-linear trend found in this study may be indicative of polydipsia experienced with poor (yet undiagnosed) glycaemic control. As we excluded those with T2D, this effect may not have been present in our analysis, providing a clearer linear trend, supporting the findings of Carroll *et al*.^(^ ^12^ ^)^, who also found a linear association between lower plain water intake and higher T2D risk. Pan *et al*.^(^ ^11^ ^)^ specifically studied women and found no association, which is also in agreement with our sex-stratified analyses.

It is unclear why a sex difference may occur. A potential explanation of these results is changes during the menstrual cycle as hormones can promote fluid retention during the luteal phase^(^ ^36^ ^)^. Speculatively, if fluid is being retained, blood volume may not increase to the same extent as it does in men, leading to a higher blood glucose concentration (Fig. 1). In addition, increases in oestradiol during the menstrual cycle lowers the osmoregulation operating point of AVP^(^ ^37^ ^)^. As AVP is a key mechanism in which hydration status may impact glycaemia, changes to its homoeostatic set-points throughout the menstrual cycle should be further investigated, particularly in relation to how plain water intake may impact these fluctuations. However, it is worth noting that approximately 40 % of females in these analyses were aged >50 years, and therefore may be (post-) menopausal. Other theories should therefore also be explored in future studies in order to understand the underlying mechanism or establish whether the finding is spurious.

Our substitution analyses of SSB and fruit juice do not support longitudinal analyses from the USA (NHS-II)^(^ ^11^ ^)^ or the UK (European Prospective Investigation into Cancer (EPIC) Norfolk)^(^ ^10^ ^)^. The discordant findings may be due to several factors. Differences in dietary assessment may provide an explanation; however, both the EPIC study and the NDNS analysed in this study used the diet diary method. Intakes of SSB may also explain the differences in findings; estimated intakes (mean or median cups/d) were lower in the NHS-II (approximate mean 0·5 cups/d)^(^ ^11^ ^)^ and EPIC-Norfolk (0·3; IQR 0·2, 0·7)^(^ ^10^ ^)^ compared with the NDNS (men: 0·9; IQR 0·4, 1·6; women: 0·5; IQR 0·3, 1·0; total sample: 0·6; IQR 0·3, 1·3). However, these comparisons should be considered cautiously because of some methodological differences, such as sample size differences and data presentation differences (e.g. Pan *et al*.^(^ ^11^ ^)^ presented their data using mean intakes, which are likely to be higher than medians).

Given the generally lower estimated SSB intakes and that substitution effects were small (in Pan *et al*.^(^ ^11^ ^)^: 7 %; 95 % CI 3, 11 % for SSB; 8 %; 95 % CI 2, 13 % for fruit juice; in O’Connor *et al*.^(^ ^10^ ^)^: 14 %; 95 % CI 1, 26 % for SSB), the substantially larger sample sizes in the NHS-II (*n* 82 902) and EPIC-Norfolk (*n* 25 639) compared with this NDNS analysis (*n* 1035) were likely the main reason for the differences in the results. This is supported by our substitution analysis of substituting SSB for plain water, which also showed a non-significant 14 % reduction in the odds of HbA1c≥5·5 % in men – an effect size that is in accordance with O’Connor *et al*.

Although SSB, and to a lesser extent fruit juices, have been associated with cardiometabolic risk^(^ ^38^ ^–^ ^40^ ^)^, a recent review concluded that there were inconsistent results with regard to substituting SSB for zero/low-energy beverages in terms of T2D risk and fasting plasma glucose concentrations^(^ ^41^ ^)^. However, the review also highlighted the paucity of evidence; thus, further research should explore associations between glycaemic control and specific beverage substitutions.

Nevertheless, our findings are supported by a randomised controlled trial in which overweight and obese women substituted SSB for water, with no effect found on cardiometabolic risk factors^(^ ^42^ ^)^. Some health markers were reduced in obese subjects; however, as these were not the focus of the present study, further research should investigate differential effects in predefined weight categories. Taken together with our findings, these results suggest that adding, rather than substituting, plain water may be the more pertinent factor to consider, as potentially the increase in water intake improves hydration status, whereas substituting beverages does not change the net intake of fluid.

Our results may also offer a partial explanation regarding findings from studies of other beverages. Generally, beverages that contribute to euhydration^(^ ^43^ ^,^ ^44^ ^)^ are associated with lower T2D risk, for example, coffee^(^ ^44^ ^,^ ^45^ ^)^, milk^(^ ^46^ ^)^ and moderate alcohol consumption^(^ ^47^ ^)^. Studies that have found a positive association between SSB and T2D often attribute the relationship to the rapidly absorbed sugar load provided by the beverages. However, depending on their solute concentration, SSB may contribute to either hydration^(^ ^43^ ^)^ or dehydration^(^ ^48^ ^)^, providing a further potential mechanism to consider when exploring the relationship between SSB and T2D.

It is unclear from the study design whether the relationship found between plain water intake and HbA1c is causal, and therefore what (if any) mechanisms highlighted in Fig. 1 underlie the association. There was no evidence of mediating effects from adding EI or fibre intake into the models, suggesting that plain water intake is independent of these factors and may act on HbA1c via other pathways. As AVP is a key blood pressure-regulating hormone, the higher systolic blood pressure in those with HbA1c≥5·5 % compared with <5·5 % support the role of AVP in the association between plain water intake and HbA1c (Fig. 1).

Plain water consumption was associated with other factors that may benefit health. For example, higher plain water intake was associated with lower water intake from other beverages, and increased fibre (in men), potentially suggestive of a healthier or less energy-dense diet. This is further supported by men with HbA1c<5·5 % having a greater contribution of total water from food sources, indicative of a lower energy-dense diet. This may explain the sex differences found in these analyses. Finally, missing data analysis showed some differences, which may suggest that more health conscious participants were more willing to provide a blood sample. Most notably, participants included in these analyses were on average more physically active than excluded respondents. Included women were also less likely to smoke, have better education and have higher total water intake than excluded women.

### Strengths and limitations

This study analysed a large sample of adults in the UK using an objective measure of long-term glycaemia and unweighed food diaries in order to assess diet. Estimated food diaries were chosen in order to reduce participant burden while still providing valid and reliable data (particularly compared with more common measures such as FFQ). Food diaries have been shown to more accurately record EI than questionnaires or 24-h recalls^(^ ^49^ ^)^, making the dietary data used in this study more accurate than most previous studies investigating water intake and T2D risk. Nonetheless, subjective measures always carry some degree of error. In the present analyses, under-reporting was controlled for by inclusion in the regression models. This did not meaningfully alter the results, suggesting that reporting of EI did not have a significant impact on the relationship between plain water intake and HbA1c.

Causality cannot be inferred from observational data, but the sample used (which included men and women from a range of ages) improves the generalisability of our findings to the UK. To our knowledge, this is the first analysis of UK data to directly investigate the relationship between HbA1c and plain water intake, building substantially on previous research in the UK^(^ ^12^ ^)^, while supporting findings from the USA^(^ ^11^ ^)^ and France^(^ ^4^ ^)^, increasing the external validity of the research.

A key limitation of any cross-sectional study is the potential for reverse causation. The exclusion of respondents with a T2D diagnosis is likely to have somewhat reduced the possibility of reverse causality (as this group may suffer from polydipsia (excessive thirst) and/or they may have made positive lifestyle changes after diagnosis), although this explanation cannot be fully excluded. Furthermore, there are issues of residual confounding. Although many variables were included in the regression models, there was limited availability of some covariates (namely PA and blood pressure), potentially affecting the power to detect strong evidence of an association. It is also unclear whether any unmeasured confounding factors would have altered the associations found.

As there is a paucity of research in this area, it is important to find plausible relationships between exposures and outcomes in order to justify experimental research. We aimed to test whether EI or fibre mediated any relationship found, as plain water intake may attenuate EI, leading to lower or less-frequent rises in blood glucose concentration^(^ ^3^ ^,^ ^50^ ^)^, and as theoretically water bulks fibre, which can lower the postprandial glycaemic response^(^ ^51^ ^)^. Although we found no evidence of these factors mediating the relationship, the findings presented have provided some interesting results with potential sex differences, which should be investigated further in randomised controlled trials.

### Conclusions

This cross-sectional analysis of 1035 NDNS respondents’ data found that plain water intake was associated with lower HbA1c in men, but not women, after controlling for a wide range of confounding factors. However, it should be noted that the reduction in HbA1c found per cup/d of plain water was not clinically meaningful. None of the substitutions modelled were associated with a change of risk in HbA1c ≥5·5 %, suggesting that the addition of plain water may be more pertinent than displacing other beverages. Longitudinal and experimental studies should be conducted in order to determine the role of plain water intake on cardiometabolic risk. Furthermore, randomised control trials should elucidate on whether the sex differences found in these analyses were genuine, as well as develop an understanding of any underlying mechanisms.

## Supplementary material

For supplementary material accompanying this paper visit http://dx.doi.org/10.1017/S0007114516003688. click here to view supplementary material
