# Supplementary material for: An investigation into the relationship between plain water intake and glycated Hb (HbA1c): a sex-stratified, cross-sectional analysis of the UK National Diet and Nutrition Survey (2008–2012)
Source: Br J Nutr. 2016 Nov 10;116(10):1770–80. doi: 10.1017/S0007114516003688 (PMC5197923; doi:10.1017/S0007114516003688)
Supplement: Supplementary file 1 [file S0007114516003688sup001.docx]

Table S1: Characteristics of male NDNS respondents in this sample according to HbA1c (n = 456)

| Characteristics |  | | HbA1c | | | | | | |
| --- | --- | --- | --- | --- | --- | --- | --- | --- | --- |
|  | Total | *SD* or *IQR* | < 5.5 % | *SD* or *IQR* | 5.5–6.49 % | *SD* or *IQR* | ≥ 6.5 % | *SD* or *IQR* | *p* for difference |
| Participants (n) | 456 | - | 221 | - | 224 | - | 11 | - | - |
| Ethnicity (% white) | 93 | - | 94 | - | 93 | - | 91 | - | 0.817 |
| Age (mean y) | 44 | 19 | 37 | 16 | 50 | 18 | 67 | 16 | < 0.001^bcd^ |
| Current smoker (%) | 22 | - | 20 | - | 25 | - | 18 | - | 0.411 |
| BMI (mean kg/m^2^) | 26.7 | 4.6 | 25.5 | 3.9 | 27.7 | 4.9 | 29.4 | 4.2 | < 0.001^bc^ |
| Education (% degree or more) | 23 | - | 23 | - | 23 | - | 9 | - | 0.547 |
| EI (mean kcal) | 2148 | 593 | 2177 | 596 | 2120 | 601 | 2122 | 349 | 0.589 |
| EI:EER (mean) | 0.73 | 0.23 | 0.74 | 0.24 | 0.72 | 0.23 | 0.78 | 0.23 | 0.605 |
| Fibre (mean g/1000 kcal) | 7 | 2 | 7 | 2 | 7 | 2 | 8 | 3 | 0.649 |
| Participants (n) | 258 | - | 111 | - | 140 | - | 7 | - | - |
| Systolic blood pressure (mean mmHg) | 130 | 14 | 128 | 12 | 131 | 14 | 143 | 17 | 0.011^c^ |
| MVPA (median h/d)^a^ | 1.2 | 0.5, 3.1 | 1.3 | 0.5, 3.1 | 1.1 | 0.5, 2.8 | 2.4 | 0.7, 5.8 | 0.485 |

*Abbreviations:* NDNS, National Diet and Nutrition Survey; HbA1c, glycated haemoglobin; *SD,* standard deviation; *IQR*, interquartile range; BMI, body mass index; EI, energy intake; EER, estimated energy requirement; MVPA, moderate to vigorous physical activity

HbA1c categories defined as: low cardiometabolic risk < 5.5 %^23-25^; increased cardiometabolic risk 5.5-6.49 %^23-25^; in the diabetic range ≥ 6.5 %^22^)

Differences calculated using ANOVA with post-hoc Bonferroni correction unless otherwise stated

**^a^** Differences calculated using Kruskal-Wallis with post-hoc Dunn-Bonferroni correction

^b^ Significantly different between < 5.5 % and 5.5–6.49 % (*p* < 0.001)

^c^ Significantly different between < 5.5 % and ≥ 6.5 % (*p* ≤ 0.016)

^d^ Significantly different between 5.5–6.49 % and ≥ 6.5 % (*p* = 0.014)

Table S2: Characteristics of female NDNS respondents in this sample according to HbA1c (n = 579)

| Characteristics |  | | HbA1c | | | | | | |
| --- | --- | --- | --- | --- | --- | --- | --- | --- | --- |
|  | Total | *SD* or *IQR* | < 5.5 % | *SD* or *IQR* | 5.5–6.49 % | *SD* or *IQR* | ≥ 6.5 % | *SD* or *IQR* | *p* for difference |
| Participants (n) | 579 | - | 295 | - | 275 | - | 9 | - | - |
| Ethnicity (% white) | 93 | - | 93 | - | 92 | - | 78 | - | 0.242 |
| Age (mean y) | 46 | 18 | 38 | 15 | 54 | 17 | 57 | 12 | < 0.001^bc^ |
| Current smoker (%) | 18 | - | 18 | - | 17 | - | 22 | - | 0.828 |
| BMI (mean kg/m^2^) | 27.0 | 5.9 | 26.1 | 5.7 | 27.7 | 5.8 | 32.8 | 8.1 | < 0.001^bcd^ |
| Education (% degree or more) | 23 | - | 26 | - | 20 | - | 100 | - | 0.068 |
| EI (mean kcal) | 1612 | 424 | 1623 | 425 | 1607 | 421 | 1403 | 434 | 0.296 |
| EI:EER (mean) | 0.75 | 0.23 | 0.75 | 0.23 | 0.76 | 0.23 | 0.64 | 0.19 | 0.294 |
| Fibre (mean g/1000 kcal) | 8 | 3 | 8 | 3 | 9 | 3 | 7 | 2 | 0.022^b^ |
| Participants (n) | 336 | - | 168 | - | 161 | - | 7 | - | - |
| Systolic blood pressure (mean mmHg) | 122 | 17 | 118 | 15 | 126 | 17 | 119 | 11 | < 0.001^b^ |
| MVPA (median h/d)^a^ | 0.6 | 0.3, 1.3 | 0.6 | 0.2, 1.4 | 0.6 | 0.3, 1.3 | 0.4 | 0.4, 0.8 | 0.421 |

*Abbreviations:* NDNS, National Diet and Nutrition Survey; HbA1c, glycated haemoglobin; *SD,* standard deviation; *IQR*, interquartile range; BMI, body mass index; EI, energy intake; EER, estimated energy requirement; MVPA, moderate to vigorous physical activity

HbA1c categories defined as: low cardiometabolic risk < 5.5 %^23-25^; increased cardiometabolic risk 5.5-6.49 %^23-25^; in the diabetic range ≥ 6.5 %^22^)

Differences calculated using ANOVA with post-hoc Bonferroni correction unless otherwise stated

**^a^** Differences calculated using Kruskal-Wallis with post-hoc Dunn-Bonferroni correction

^b^ Significantly different between < 5.5 % and 5.5–6.49 % (*p* ≤ 0.033)

^c^ Significantly different between < 5.5 % and ≥ 6.5 % (*p* = 0.002)

^d^ Significantly different between 5.5–6.49 % and ≥ 6.5 % (*p* = 0.030)

Table S3: Linear regression analysis of cups/d of plain water on HbA1c in men (n = 258) and women (n = 336) with the inclusion of systolic blood pressure and physical activity as covariates

|  |  | Men |  |  | Women |  |
| --- | --- | --- | --- | --- | --- | --- |
| Model | B | 95 % CI | *p* for trend | B | 95 % CI | *p* for trend |
| 1 | -0.04 | -0.07, -0.02 | 0.002 | -0.03 | -0.05, 0.00 | 0.545 |
| 2 | -0.02 | -0.04, 0.00 | 0.006 | -0.01 | -0.03, 0.02 | 0.644 |
| 3 | -0.03 | -0.05, -0.01 | 0.002 | -0.01 | -0.03, 0.02 | 0.606 |
| 4 | -0.03 | -0.05, -0.01 | 0.002 | -0.01 | -0.03, 0.02 | 0.654 |
| 5 | -0.03 | -0.05, -0.01 | 0.003 | -0.01 | -0.03, 0.02 | 0.661 |
| 6 | -0.03 | -0.05, -0.01 | 0.003 | -0.01 | -0.03, 0.02 | 0.661 |
| 7 | -0.03 | -0.05, -0.01 | 0.003 | -0.01 | -0.03, 0.02 | 0.655 |

*Abbreviations*: HbA1c, glycated haemoglobin; CI, confidence interval

*p* for interaction = 0.319 for HbA1c and water intake between sexes

B value represents the change in HbA1c per increase of one cup/d of water

Model 1 = Plain water

Model 2 = Model 1 + age, ethnic group, BMI, smoking status, qualifications (degree or not)

Model 3 = Model 2 + Total drinks minus plain water

Model 4 = Model 3 + energy intake, reporter category

Model 5 = Model 4 + Fibre (g/1000kcal)

Model 6 = Model 5 + systolic blood pressure

Model 7 = Model 6 + moderate to vigorous physical activity (h/d)

Table S4: Logistic regression of cups/d of water on HbA1c ≥ 5.5 % compared to < 5.5 % in men (n = 258) and women (n = 336)

|  | Men | | Women | |
| --- | --- | --- | --- | --- |
| Model | OR | 95 % CI | OR | 95% CI |
| 1 | 0.85 | 0.74, 0.97 | 0.86 | 0.75, 0.98 |
| 2 | 0.90 | 0.77, 1.05 | 0.91 | 0.78, 1.06 |
| 3 | 0.86 | 0.73, 1.01 | 0.92 | 0.78, 1.07 |
| 4 | 0.85 | 0.72, 1.07 | 0.91 | 0.78, 1.07 |
| 5 | 0.87 | 0.74, 1.03 | 0.92 | 0.78, 1.07 |
| 6 | 0.87 | 0.74, 1.03 | 0.92 | 0.78, 1.07 |
| 7 | 0.87 | 0.74, 1.04 | 0.92 | 0.78, 1.07 |

*Abbreviations*: HbA1c, glycated haemoglobin; OR, odds ratio; CI, confidence interval

*p* for interaction = 0.858 for HbA1c and water intake between sexes

Model 1 = Plain water

Model 2 = Model 1 + age, ethnic group, BMI, smoking status, qualifications (degree or not)

Model 3 = Model 2 + Total drinks minus plain water

Model 4 = Model 3 + energy intake, reporter category

Model 5 = Model 4 + Fibre (g/1000kcal)

Model 6 = Model 5 + systolic blood pressure

Model 7 = Model 6 + moderate to vigorous physical activity (h/d)

Table S5: Substitution models in men (n = 456) and women (n = 579), modelling the odds of having HbA1c ≥ 5.5 % compared to ≤ 5.5 %

|  | Men | | Women | |
| --- | --- | --- | --- | --- |
| Substitution | OR | 95 % CI | OR | 95 % CI |
| SSBs for plain water | 0.86 | 0.67, 1.11 | 0.90 | 0.66, 1.23 |
| Fruit juice for plain water | 1.11 | 0.67, 1.83 | 0.74 | 0.40, 1.36 |
| ASBs for plain water | 0.96 | 0.77, 1.21 | 0.83 | 0.66, 1.05 |
| SSBs for ASBs | 0.89 | 0.67, 1.18 | 1.08 | 0.76, 1.54 |

*Abbreviations:* HbA1c, glycated haemoglobin; OR, odds ratio; CI, confidence interval; ASBs, artificially sweetened beverages; SSBs, sugar-sweetened beverages

Table S6: Characteristics of excluded male NDNS respondents according to plain water intake (n = 584)

| Characteristics |  | | Plain water intake | | | | | |  |
| --- | --- | --- | --- | --- | --- | --- | --- | --- | --- |
|  | Total | *SD* or *IQR* | ≤ 0.21 cups/d | *SD* or *IQR* | 0.22-1.59 cups/d | *SD* or *IQR* | > 1.59 cups/d | *SD* or *IQR* | *p* for difference |
| Participants (n) | 584 | - | 205 | - | 191 | - | 188 | - | - |
| Ethnicity (% white) ^†^ | 89 | - | 95 | - | 87 | - | 85 | - | < 0.001 |
| Age (mean y) | 44 | 20 | 46 | 22 | 45 | 21 | 40 | 17 | 0.004^bc^ |
| EI (mean kcal) ^†^ | 2055 | 602 | 2049 | 606 | 1966 | 550 | 2152 | 636 | 0.010^c^ |
| Fibre (mean g/1000 kcal) | 7 | 2 | 7 | 2 | 7 | 3 | 8 | 3 | < 0.001^bd^ |
| Participants (n) | 578 | - | 200 | - | 191 | - | 187 | - | ^-^ |
| Current smoker (%) | 26 | - | 36 | - | 22 | - | 20 | - | < 0.001 |
|  |  |  |  |  |  |  |  |  |  |
| Participants (n) | 572 | - | 201 | - | 189 | - | 182 | - | - |
| Education (% degree or more) | 20 | - | 14 | - | 20 | - | 27 | - | 0.005 |
|  |  |  |  |  |  |  |  |  |  |
| Participants | 518 | - | 177 | - | 168 | - | 173 | - | - |
| EI:EER (mean) | 0.72 | 0.24 | 0.72 | 0.25 | 0.69 | 0.23 | 0.74 | 0.25 | 0.229 |
|  |  |  |  |  |  |  |  |  |  |
| Participants (n) | 502 | - | 172 | - | 164 | - | 166 | - | - |
| BMI (mean kg/m^2^) | 26.6 | 4.9 | 26.8 | 5.2 | 26.5 | 5.0 | 26.6 | 4.6 | 0.872 |
| Participants (n) | 239 | - | 76 | - | 72 | - | 91 | - | - |
| Systolic blood pressure (mean mmHg) | 128 | 15 | 128 | 14 | 129 | 16 | 129 | 14 | 0.912 |
| Participants (n) | 383 | - | 131 | - | 130 | - | 122 | - | - |
| MVPA (median h/d)^a†^ | 0.8 | 0.3, 2.5 | 0.5 | 0.1, 2.1 | 0.9 | 0.3, 2.9 | 1.2 | 0.5, 2.8 | 0.002^bd^ |

*Abbreviations*: NDNS, National Diet and Nutrition Survey; *SD,* standard deviation; *IQR*, interquartile range; EI, energy intake; EER, estimated energy requirement; BMI, body mass index; MVPA, moderate to vigorous physical activity. 1 cup = 240mL

Differences calculated using ANOVA with post-hoc Bonferroni correction unless otherwise stated

**^a^** Differences calculated using Kruskal-Wallis with post-hoc Dunn-Bonferroni correction

^b^ Significantly different between ≤ 0.21 cups/d and > 1.59 cups/d (*p* ≤ 0.007)

^c^ Significantly different between 0.22-1.59 cups/d and > 1.59 cups/d (*p* ≤ 0.020)

^d^ Significantly different between < 0.21 cups/d and 0.22-1.59 cups/d (*p* ≤ 0.041)

**^†^** Significant difference between included and excluded respondents: Included men were more likely to be white (93 % compared to 89 %; *P* = 0.023); excluded men consumed 93 kcal/d fewer than included men (*p* = 0.013); excluded men did 0.4 h/d less MVPA than included men (*P* = 0.002)

Table S7: Characteristics of excluded female NDNS respondents according to plain water intake (n = 756)

| Characteristics |  | | Plain water intake | | | | | |  |
| --- | --- | --- | --- | --- | --- | --- | --- | --- | --- |
|  | Total | *SD* or *IQR* | ≤ 0.66 cups/d | *SD* or *IQR* | 0.67-2.05 cups/d | *SD* or *IQR* | > 2.05 cups/d | *SD* or *IQR* | *p* for difference |
| Participants (n) | 756 | - | 287 | - | 238 | - | 231 | - | - |
| Ethnicity (% white) | 90 | - | 96 | - | 86 | - | 86 | - | < 0.001 |
| Age (SD)^†^ | 44 | 20 | 44 | 21 | 44 | 21 | 43 | 20 | 0.946 |
| EI (mean kcal) ^†^ | 1545 | 432 | 1536 | 418 | 1574 | 471 | 1528 | 406 | 0.458 |
| Fibre (mean g/1000 kcal) | 8 | 3 | 8 | 2 | 8 | 3 | 9 | 3 | < 0.001^bcd^ |
|  |  |  |  |  |  |  |  |  |  |
| Participants (n) | 745 | - | 282 | - | 236 | - | 227 | - | - |
| Education (% degree or more) ^†^ | 18 | - | 13 | - | 21 | - | 20 | - | 0.021 |
|  |  |  |  |  |  |  |  |  |  |
| Participants (n) | 660 | - | 253 | - | 202 | - | 205 | - | - |
| EI:EER (mean) | 0.74 | 0.24 | 0.74 | 0.23 | 0.75 | 0.26 | 0.73 | 0.24 | 0.611 |
|  |  |  |  |  |  |  |  |  |  |
|  |  |  |  |  |  |  |  |  |  |
| Participants (n) | 749 | - | 283 | - | 237 | - | 229 | - | - |
| Current smoker (%)^†^ | 22 | - | 29 | - | 19 | - | 17 | - | 0.001 |
|  |  |  |  |  |  |  |  |  |  |
| Participants (n) | 643 | - | 248 | - | 196 | - | 199 | - | - |
| BMI (mean kg/m^2^) | 27.1 | 6.1 | 27.1 | 5.7 | 27.3 | 6.5 | 26.9 | 6.4 | 0.827 |
|  |  |  |  |  |  |  |  |  |  |
| Participants (n) | 307 | - | 110 | - | 91 | - | 106 | - | - |
| Systolic blood pressure (mean mmHg) | 123 | 18 | 124 | 19 | 123 | 17 | 121 | 18 | 0.564 |
|  |  |  |  |  |  |  |  |  |  |
| Participants (n) | 501 | - | 185 | - | 158 | - | 158 | - | - |
| MVPA (median h/d)^a†^ | 0.4 | 0.1, 1.2 | 0.3 | 0.1, 0.8 | 0.5 | 0.2, 1.1 | 0.8 | 0.3, 1.5 | < 0.001^bc^ |

*Abbreviations*: NDNS, National Diet and Nutrition Survey; *SD,* standard deviation; *IQR*, interquartile range; EI, energy intake; EER, estimated energy requirement; BMI, body mass index; MVPA, moderate to vigorous physical activity. 1 cup = 240mL

Differences calculated using ANOVA with post-hoc Bonferroni correction unless otherwise stated

**^a^** Differences calculated using Kruskal-Wallis with post-hoc Dunn-Bonferroni correction

^b^ Significantly different between ≤ 0.66 cups/d and 0.67-2.05 cups/d (*p* ≤ 0.044)

^c^ Significantly different between ≤ 0.66 cups/d and > 2.05 cups/d (*p* < 0.001)

^d^ Significantly different between 0.67-2.05 cups/d and > 2.05 cups/d (*p* = 0.001)

**^†^**Significant difference between included and excluded respondents: Excluded women were 2.4 y younger than included women (*p* = 0.022); excluded women consumed 67 kcal/d fewer than included women (*p* = 0.005); excluded women did 0.2 h/d less MVPA than included women (*P* = 0.001); excluded women were more likely to have a degree or higher than included women (18 % compared to 23 %; *P* = 0.026); excluded women were more likely to be a current smoker than included women (18 % compared to 22 %; *P* = 0.027)

Table S8: Water from difference sources and association with plain water intake in excluded respondents

| Beverage type | Men (n = 584) | | | | Women (n = 756) | | | |
| --- | --- | --- | --- | --- | --- | --- | --- | --- |
|  | Consumers (%)^a^ | Median intake^a^ | *IQR* | Correlation with water intake | Consumers (%)^a^ | Median intake^a^ | *IQR* | Correlation with water intake |
| Plain water | 73 | 1.4 | 0.6, 2.8 | - | 86 | 1.4 | 0.6, 2.6 | - |
| Alcoholic^‡^ drinks (cups/d) | 57 | 1.7 | 0.8, 3.3 | -0.06 | 45 | 0.5 | 0.2, 1.0 | -0.04 |
| ASBs (cups/d) | 31 | 0. 9 | 0.5, 1.7 | -0.02 | 36 | 0.7 | 0.3, 1.4 | -0.14 |
| Fruit juice (cups/d) | 37 | 0.4 | 0.2, 0.7 | 0.04 | 40 | 0.3 | 0.2, 0.6 | 0.01 |
| Milk (cups/d) ^‡^ | 93 | 0.5 | 0.3, 0.9 | -0.05 | 92 | 0.4 | 0.2, 0.8 | -0.07 |
| SSBs (cups/d) | 53 | 0.9 | 0.5, 1. 7 | -0.13 | 49 | 0.6 | 0.3, 1.2 | -0.21 |
| Tea/coffee (cups/d) ^†‡^ | 87 | 2.6 | 1.4, 3.9 | -0.23 | 87 | 2.6 | 1.5, 3. 9 | -0.16 |
| Miscellaneous beverages (cups/d) | 14 | 0.3 | 0.2, 0.7 | 0.06 | 23 | 0.4 | 0.2, 0. 8 | -0.08 |
| Total beverages^b^ (cups/d) ^‡^ | 100 | 5.7 | 3.7, 7.4 | -0.20 | 100 | 4.3 | 3.0, 5.7 | -0.23 |
| Water from food (g/d) | 100 | 563 | 432, 711 | 0.22 | 100 | 525 | 396, 655 | 0.26 |
| Total water (g/d)^b‡^ | 100 | 1942 | 1414, 2485 | -0.12 | 100 | 1618 | 1194, 1961 | -0.12 |

*Abbreviations*: *IQR*, interquartile range; ASBs, artificially sweetened beverages; SSBs, sugar-sweetened beverages

1 cup = 240 mL

Correlation using Spearman’s rho

^a^ Percent of sample who reported consuming each beverage type; median intakes are of consumers only

^b^ Total minus plain water

**^†^** Significantly more consumed by included men than excluded men (*P* = 0.040)

**^‡^** Significantly more consumed by included women than excluded women (*P* ≤ 0.011)
